# Structural and Biochemical Analysis of the Furan Aldehyde Reductase YugJ from *Bacillus subtilis*

**DOI:** 10.3390/ijms23031882

**Published:** 2022-02-08

**Authors:** Hye Yeon Cho, Mi Sun Nam, Ho Jeong Hong, Wan Seok Song, Sung-il Yoon

**Affiliations:** 1 Division of Biomedical Convergence, College of Biomedical Science, Kangwon National University, Chuncheon 24341, Korea; jhy013@naver.com (H.Y.C.); assdec@naver.com (M.S.N.); in_sglr@naver.com (H.J.H.); 2 Institute of Bioscience and Biotechnology, Kangwon National University, Chuncheon 24341, Korea

**Keywords:** *Bacillus subtilis*, YugJ, crystal structure, furan aldehyde reductase, 5-hydroxymethylfurfural, NADPH cofactor, Ni^2+^ cofactor

## Abstract

NAD(H)/NADP(H)-dependent aldehyde/alcohol oxidoreductase (AAOR) participates in a wide range of physiologically important cellular processes by reducing aldehydes or oxidizing alcohols. Among AAOR substrates, furan aldehyde is highly toxic to microorganisms. To counteract the toxic effect of furan aldehyde, some bacteria have evolved AAOR that converts furan aldehyde into a less toxic alcohol. Based on biochemical and structural analyses, we identified *Bacillus subtilis* YugJ as an atypical AAOR that reduces furan aldehyde. YugJ displayed high substrate specificity toward 5-hydroxymethylfurfural (HMF), a furan aldehyde, in an NADPH- and Ni^2+^-dependent manner. YugJ folds into a two-domain structure consisting of a Rossmann-like domain and an α-helical domain. YugJ interacts with NADP and Ni^2+^ using the interdomain cleft of YugJ. A comparative analysis of three YugJ structures indicated that NADP(H) binding plays a key role in modulating the interdomain dynamics of YugJ. Noticeably, a nitrate ion was found in proximity to the nicotinamide ring of NADP in the YugJ structure, and the HMF-reducing activity of YugJ was inhibited by nitrate, providing insights into the substrate-binding mode of YugJ. These findings contribute to the characterization of the YugJ-mediated furan aldehyde reduction mechanism and to the rational design of improved furan aldehyde reductases for the biofuel industry.

## 1. Introduction

NAD(H)/NADP(H)-dependent aldehyde/alcohol oxidoreductase (AAOR) catalyzes aldehyde reduction or alcohol oxidation [1]. AAOR is ubiquitously found in organisms from prokaryotes to eukaryotes and employed for diverse physiological purposes ranging from metabolic processes to cellular defense against exogenous toxic aldehydes or alcohols [2,3]. In addition, AAOR has recently been reported to contribute to oxidative stress relief, biofilm formation, and virulence [4,5,6,7]. AAORs can be classified into three groups based on metallic cofactor and molecular size: group I, zinc-dependent medium-chain AAORs (~350 residues); group II, zinc-independent short-chain AAORs (~250 residues); and group III, iron-containing AAORs (~385 residues) [8].

Group III AAORs typically coordinate an Fe^2+^ metallic cofactor using highly conserved histidine and aspartic residues. However, some group III AAORs require other divalent metal ions, such as Zn^2+^, Ni^2+^, Mg^2+^, Cu^2+^, Co^2+^, and Mn^2+^, for oxidoreduction activity [9,10,11,12,13,14,15]. Furthermore, each AAOR exhibits a unique substrate specificity depending on its physiological role, although it generally reacts with multiple types of aldehydes or alcohols [13,16,17]. For example, both *Escherichia coli* YqhD and *Thermotoga hypogea* AAOR were active toward a wide range of aldehydes [16,18]. *E. coli* YqhD showed the highest activity for butyraldehyde but little activity for acetaldehyde. In contrast, *T. hypogea* AAOR was highly active against acetaldehyde. Because group III AAORs display different cofactor and substrate specificities, they should be individually studied to reveal their enzymatic properties and physiological functions.

Among the substrates of group III AAORs, furan aldehydes, including furfural and 5-hydroxymethylfurfural (HMF), are highly toxic to microorganisms because they induce accumulation of reactive oxygen species, DNA damage, sugar metabolism inhibition, and translational repression [19,20,21,22]. To control the toxicity of furan aldehydes, some microorganisms have been reported to reduce furan aldehydes to less toxic alcohols with AAOR [23,24,25,26,27]. Because of its detoxification activity, furan aldehyde-reducing AAOR can be applied to the biofuel industry, in which fuel ethanol is obtained via the microbial fermentation of lignocellulosic biomass [28,29]. For effective, economical fermentation, it is necessary to extract fermentable sugars from lignocellulosic biomass through pretreatment processes, including high temperature and acid treatment [30]. However, the pretreatment processes generate undesirable toxic byproducts that inhibit the growth of ethanologenic bacteria and reduce ethanol yield. Because furan aldehydes are the most toxic byproducts, furan aldehyde-eliminating strategies, such as AAOR-mediated reduction of furan aldehydes, are required to improve ethanol production. To employ AAORs in the biofuel industry and understand the mechanism for furan aldehyde detoxification, furan aldehyde-reducing AAORs should be studied through structural and biochemical analyses.

*Bacillus subtilis* is a Gram-positive soil bacterium that has broad industrial applications because of its diverse industry-friendly features, such as a wide range of carbohydrate catabolic pathways, extensive protein secretion, and industrial safety [31,32,33]. In addition, *B. subtilis* can withstand high temperatures, high ethanol concentrations, and low pH [34]. Therefore, *B. subtilis* has been considered a biocatalyst for generating valuable compounds from lignocellulosic biomass. Recently, genetic manipulation-based attempts to develop ethanologenic *B. subtilis* strains have been reported [35,36]. However, it is unclear whether *B. subtilis* expresses furan aldehyde-reducing AAOR to mediate cellular tolerance to furan aldehydes. Based on structural and biochemical studies, we report here that YugJ from *B. subtilis* is an atypical NADPH-dependent group III AAOR that reduces furan aldehyde in the presence of a Ni^2+^ cofactor. Furthermore, our inhibition assays and molecular docking analysis suggest that YugJ interacts with the HMF substrate in proximity to NADPH and Ni^2+^ in an interdomain cleft.

## 2. Results and Discussion

### 2.1. Aldehyde Reductase Activity of YugJ

**Figure 1:**
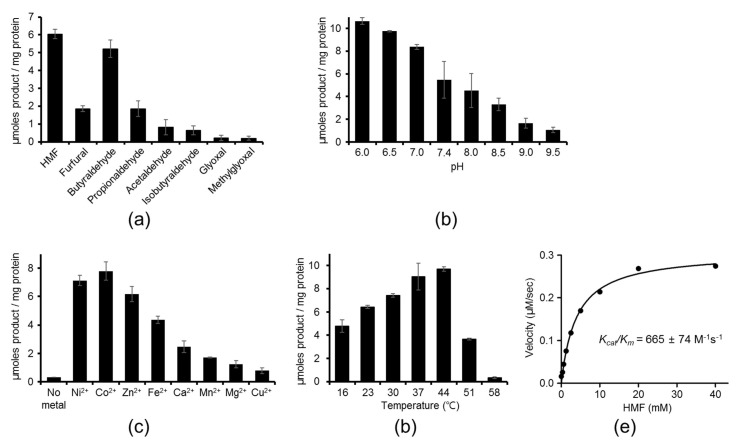
Catalytic properties of YugJ. (**a**) Substrate specificity of YugJ as an aldehyde reductase. The catalytic activity of recombinant YugJ protein toward diverse aldehydes was determined in the presence of NADPH and Ni^2+^ at pH 7.4 by monitoring NADPH oxidation, which was measured by a decrease in 340 nm absorbance. (**b**) pH dependence of YugJ-mediated catalysis. The HMF reduction activity of YugJ was determined at different pH values in the presence of NADPH and Ni^2+^. (**c**) Metal dependence of YugJ-mediated catalysis. The HMF reduction activity of YugJ was determined in the absence or presence of a divalent metal ion at pH 7.4 with NADPH. (**d**) Temperature dependence of YugJ-mediated catalysis. The HMF reduction activity was determined at different temperatures at pH 7.4 in the presence of NADPH and Ni^2+^. (**e**) Catalytic efficiency of the HMF reductase YugJ derived from the Michaelis–Menten curve. The enzymatic activity of YugJ was determined at different HMF concentrations in the presence of NADPH and Ni^2+^ at pH 7.4.

As the first step to investigate the catalytic characteristics of YugJ, we analyzed the substrate specificity of YugJ. The reductase activity of recombinant YugJ protein was determined using an array of aldehydes as a putative substrate in the presence of NADPH as a reducing cofactor. YugJ exhibited the highest reductase activity for HMF, in which an aldehyde group is attached to the hydroxymethylated furan ring (Figure 1a). YugJ was also catalytically active for furfural but had lower activity than for HMF. Although YugJ actively catalyzed HMF reduction, YugJ did not drive the reverse reaction. Essentially no enzymatic activity was detected with 2,5-bis(hydroxymethyl)furan, an alcohol counterpart of HMF, in the presence of NADP (Supplementary Figure S1). These observations indicate that YugJ functions as a furan aldehyde reductase rather than as an alcohol dehydrogenase.

In addition to its activity against cyclic aldehydes, YugJ reduced linear aldehydes. Among butyraldehyde, propionaldehyde, and acetaldehyde, YugJ displayed the highest activity for butyraldehyde, a linear four-carbon aldehyde, at a level comparable to that for HMF. In contrast to butyraldehyde, low activity was observed for isobutyraldehyde, a branched four-carbon aldehyde. This substrate length and shape dependence of YugJ activity has also been reported for other aldehyde reductases, such as *E. coli* YqhD, a close homolog of YugJ [16]. As observed for YugJ, YqhD showed high specificity for butyraldehyde among linear aldehydes. However, YqhD differs from YugJ in furan aldehyde reduction activity. YugJ displayed substantial activities for HMF and furfural, whereas extremely low activity was observed for YqhD [37]. Another difference in substrate specificity was identified for α-oxoaldehydes, including glyoxal and methylglyoxal, which are generated by oxidative stress and cause cellular damage [5]. *E.* *coli* employs YqhD to evade the toxic effects of glyoxal and methylglyoxal [17]. In contrast, YugJ displayed negligible activity toward glyoxal and methylglyoxal. In summary, YugJ displays a unique substrate specificity for HMF, suggesting that YugJ contributes to cellular tolerance for furan aldehydes.

To further explore the enzymatic properties of YugJ, the pH dependence of YugJ was examined in a range of pH 6.0–9.5. The catalytic activity of YugJ at pH values below 6.0 could not be addressed due to NADPH instability and the inability of YugJ to bind metals at low pH [38]. YugJ protein exhibited higher HMF-reducing activity at pH 6.0–7.0 than at alkaline pH values (Figure 1b). In addition to pH dependence, YugJ displayed metal dependence. When YugJ protein was demetallized using EDTA at pH 5.0, the metal-free YugJ protein did not reduce HMF at pH 7.4 (Figure 1c). However, when supplemented with divalent metal ions, YugJ reduced HMF. The highest catalytic activity of YugJ was observed with Ni^2+^ and Co^2+^, followed by Zn^2+^, Fe^2+^, Ca^2+^, Mn^2+^, Mg^2+^, and Cu^2+^. In general, Ni^2+^ and Co^2+^ ions exhibit similar coordination properties, and many Ni^2+^-coordinating proteins can use Co^2+^ as an alternative metallic cofactor [39,40]. Therefore, YugJ is expected to mediate the catalytic reaction with Co^2+^ in a manner similar to that observed with Ni^2+^. To evaluate the temperature dependence of YugJ, HMF reduction activity was measured at different temperatures (Figure 1d). The HMF reduction activity of YugJ gradually increased with increasing temperature between 16 and 44 °C and rapidly decreased above 44 °C. Negligible activity was observed at 58 °C. Furthermore, the kinetic parameters of YugJ were derived by fitting the Michaelis–Menten model to the catalytic activities of YugJ that were obtained with a wide range of HMF concentrations in the presence of NADPH and Ni^2+^ at pH 7.4, which corresponds to the pH value of the *B. subtilis* cytosol (Figure 1e) [41]. YugJ showed high catalytic efficiency (*k_cat_/K_m_*, 665 ± 74 M^−1^ sec^−1^) with a *k_cat_* value of 2.55 ± 0.08 sec^−1^ and a *K_m_* value of 3.88 ± 0.53 mM.

### 2.2. Metal Ion Recognition by YugJ

**Figure 2:**
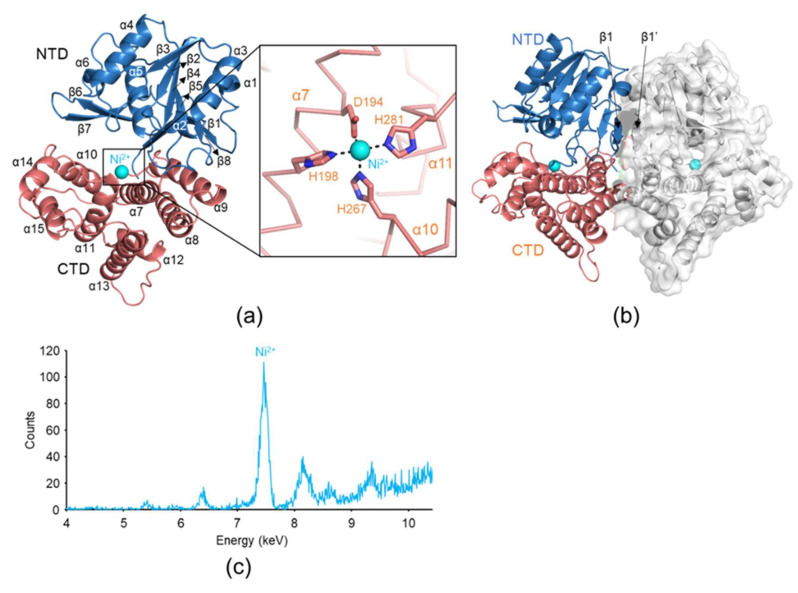
Ni^2+^ ion binding by YugJ. (**a**) Overall structure of YugJ^Ni^ and Ni^2+^ ion coordination by YugJ residues. YugJ and Ni^2+^ in the YugJ^Ni^ structure are depicted as ribbons (NTD, light blue; CTD, light red) and a sphere (cyan), respectively. Ni^2+^ ion coordination by the aspartate and histidine residues of YugJ is shown in a close-up view in the right panel. (**b**) Structure of the YugJ^Ni^ dimer. One protomer is depicted as blue and red ribbons, and the other is shown as gray ribbons with gray transparent surfaces. The Ni^2+^ ions are represented by cyan spheres. (**c**) X-ray fluorescence spectrum collected from a YugJ crystal. The peak at 7.46 keV is derived from Ni^2+^ ions in the YugJ crystal.

To obtain structural insights into YugJ-mediated aldehyde reduction, we determined the crystal structure of YugJ in complex with Ni^2+^ ions (YugJ^Ni^) at a 2.15 Å resolution in space group P2_1_2_1_2_1_ by molecular replacement (Figure 2a and Table 1). The asymmetric unit of the YugJ crystal contains two YugJ polypeptide chains (chains A and B), which are arranged into a homodimer (Figure 2b). Such a dimer was also detected in solution. In gel-filtration chromatography, YugJ protein (calculated molecular weight of a monomer, 43.3 kDa) was eluted as a dimer (apparent molecular weight, ~74 kDa) between 44 and 158 kDa protein standards (Supplementary Figure S2).

YugJ^Ni^ consists of two domains, an N-terminal domain (NTD; residues 1–183) and a C-terminal domain (CTD; residues 184–387) (Figure 2a). The NTD contains a Rossmann-like α/β fold, in which a six-stranded parallel β-sheet (β1-β8-β5-β4-β2-β3) is centrally located and surrounded by six α-helices (α1–α6). One face of the Rossmann-like fold is also decorated by the β6–β7 hairpin that protrudes from the β5 and β8 strands. The β1 strand is the most extended β-strand consisting of 11 residues and is antiparallelly aligned with its counterpart β-strand from the facing subunit, allowing YugJ to form a 12-stranded β-sheet and dimerize (Figure 2b). In contrast to the NTD, the CTD exclusively consists of α-helices and can be divided into two α-helix bundles (α7–α9 helices and α10–α15 helices). The NTD and CTD are organized to generate a cleft, which accommodates the NADP(H) cofactor (see below).

YugJ shows structural similarity to other group III AAORs, including *E. coli* YqhD (PDB ID 1OJ7), *Klebsiella pneumoniae* 1,3-propanediol dehydrogenase (PDB ID 3BFJ), and *Zymomonas mobilis* alcohol dehydrogenase 2 (PDB ID 3OWO) (Supplementary Figures S3 and S4) [12,42,43]. These group III AAORs exhibit significant sequence (sequence identity, 27%~37%) and structure (root-mean-square deviation, 1.7~2.3 Å) similarities to YugJ, indicating that YugJ is a group III AAOR. In contrast to the overall structural similarity of group III AAORs, the local structures of the α9–α10 loop are categorized into two conformations depending on length (Supplementary Figure S5). The α9–α10 loops of most group III AAORs are short and make a tight turn between the α9 and α10 helices. However, the α9–α10 loop of YugJ is longer and adopts a more extended coil structure like that of *E. coli* YqhD. Interestingly, this unique structure of the YugJ α9–α10 loop is involved in regulation of access to the active site (see below).

In the YugJ^Ni^ structure, a strong electron density peak that belongs to a metal ion was found in each YugJ monomer, although a metal ion was not used for crystallization. To define the identity of the metal ion, an X-ray fluorescence spectrum was obtained from a YugJ crystal [44]. In the spectrum, a prominent peak was observed at 7.46 keV, which corresponds to the X-ray emission energy of the Ni^2+^ ion, indicating that the metal ion observed in the YugJ structure is a Ni^2+^ ion (Figure 2c). The Ni^2+^ ion is located between the two α-helix bundles of the CTD in the interdomain cleft and coordinated by conserved aspartate (Asp194) and histidine (His198, His267, and His281) residues (Figure 2a). Given that YugJ exhibited high aldehyde reductase activity in the presence of Ni^2+^ ions and was observed with Ni^2+^ ions in the crystal structure of YugJ^Ni^, we conclude that YugJ is an atypical group III AAOR that favors a Ni^2+^ ion as a metallic cofactor.

### 2.3. NADP(H) Recognition by YugJ

**Figure 3:**
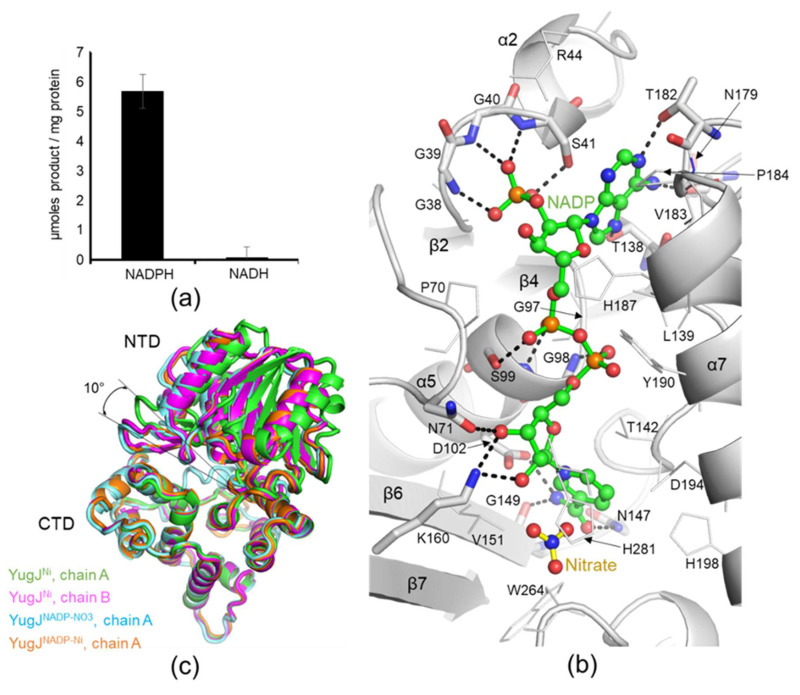
NADP recognition by YugJ. (**a**) NADPH-dependent HMF reduction by YugJ. The HMF reduction activity of YugJ was determined in the presence of NADPH or NADH as a cofactor. (**b**) NADP-binding site of YugJ in the YugJ^NADP-NO3^ structure. The NADP cofactor (carbon, green sphere; nitrogen, blue sphere; oxygen, red sphere; phosphorus, orange sphere; interatomic bond, green stick) and its neighboring nitrate ion (nitrogen, blue sphere; oxygen, red sphere; interatomic bond, yellow stick) are represented by ball-and-stick models. The NADP-binding residues of YugJ are shown as gray lines on the YugJ structure (gray ribbons). In particular, the YugJ residues that form polar interactions with NADP are depicted as gray sticks. (**c**) NADP binding-mediated restriction of YugJ interdomain flexibility. The YugJ^Ni^ (chain A, green ribbons; chain B, magenta ribbons), YugJ^NADP-NO3^ (cyan ribbons), and YugJ^NADP-Ni^ (orange ribbons) structures are superimposed based on their CTDs.

Group III AAORs use NADPH or NADH as a reducing cofactor for catalysis. To define the cofactor specificity of YugJ, the HMF-reducing activity of YugJ was measured in the presence of NADPH or NADH. YugJ was highly active as an HMF reductase when supplemented with NADPH, but it was inactive with NADH, indicating that YugJ is an NADPH-dependent aldehyde reductase (Figure 3a).

To address the specific interaction of YugJ with NADP(H), we determined the crystal structures of YugJ in complex with NADP in two crystal forms (Supplementary Figure S6 and Table 1). The first crystal structure (space group P1) contains both the NADP cofactor and Ni^2+^ ion and was named YugJ^NADP-Ni^. The second NADP-bound structure (space group P2_1_) additionally harbors nitrate ions near NADP and was named YugJ^NADP-NO3^. The YugJ^NADP-NO3^ structure exhibits well-defined electron density for each atom of NADP. However, in the YugJ^NADP-Ni^ structure, the nicotinamide ring of NADP is disordered, and only the remaining NADP region was built (Supplementary Figure S6). Therefore, the interaction between YugJ and NADP is described with the YugJ^NADP-NO3^ structure unless otherwise specified.

In the YugJ^NADP-NO3^ structure, the NADP molecule is deeply embedded into the cleft between the two domains of YugJ (Figure 3b). NADP leans toward the NTD in the interdomain cleft and exhibits more interactions with the NTD than with the CTD. Each YugJ polypeptide chain from the dimer binds one NADP molecule with a buried surface area of ~690 Å^2^, mainly via hydrogen bonds and van der Waals interactions. The adenine moiety of NADP is sandwiched between the Ser41 and Val183 residues and forms hydrogen bonds with YugJ residues from the β5–β6 loop (Thr138) and β8–α7 loop (Asn179 and Thr182). The pyrophosphate group in the middle of NADP is located in close proximity to the conserved cofactor-binding motif of aldehyde reductase enzymes (Gly96, Gly97, Gly98, and Ser99 residues; GGGS^1^ motif; Supplementary Figure S3) and is stabilized via hydrogen bonds with the Gly98 and Ser99 residues from the α5 helix. The nicotinamide ring and its linked ribose moiety in NADP also form multiple hydrogen bonds with YugJ residues. The YugJ Asn71 and Lys160 residues at the β3–α4 loop and β7 strand, respectively, interact with the hydroxyl groups of the NADP ribose moiety. The nicotinamide ring interacts with the YugJ Asp102, Asn147, and Gly149 residues from the α5 helix, β5–β6 loop, and β6 strand, respectively, in the YugJ^NADP-NO3^ structure. However, in the YugJ^NADP-Ni^ structure, the nicotinamide-YugJ interaction is not observed because of nicotinamide disorder.

The YugJ-NADP complex structure allows us to explain why YugJ prefers NADPH to NADH as a reducing cofactor. At the 2′ position of the adenine-linked ribose moiety, NADP(H) has an additional phosphate group that is not present in NAD(H). In the YugJ^NADP-NO3^ structure, the 2′-phosphate group of NADP is inserted into the curved structure of the second GGGS motif (GGGS^2^ motif; Gly38-Ser41 residues) at the β2–α2 loop (Figure 3b). The GGGS^2^ motif extensively interacts with the 2′-phosphate group of NADP using the Gly39-Ser41 main chains and the Ser41 side chain. The GGGS^2^ motif is highly conserved across NADP(H)-dependent group III AAOR enzymes (YqhD in Supplementary Figure S3) [12]. However, in NAD(H)-dependent group III AAOR enzymes, the Gly38 residue at the GGGS^2^ motif is substituted with an aspartate residue, which sterically clashes with the 2′-phosphate group of NADP(H) (*K. pneumoniae* 1,3-propanediol dehydrogenase and *Z. mobilis* alcohol dehydrogenase 2 in Supplementary Figure S3) [42]. Overall, we conclude that the conserved GGGS^2^ motif of YugJ plays a key role in distinguishing NADP(H) from NAD(H).

A comparative analysis of the NADP-free and NADP-bound YugJ structures revealed an NADP binding-mediated change in the interdomain dynamics of YugJ (Figure 3c). YugJ exhibits interdomain flexibility in the absence of NADP, given that YugJ chains A and B in the YugJ^Ni^ structure adopt open and closed conformations, respectively, which differ in interdomain angle by ~10° (Supplementary Figure S7a). However, NADP binding restricts the interdomain organization of YugJ into the closed conformation. Each protomer of the NADP-bound YugJ structures (YugJ^NADP-Ni^ chains A, B, C, and D and YugJ^NADP-NO3^ chains A and B) adopts a closed conformation with a smaller interdomain angle, similar to that of YugJ^Ni^ chain B (Figure 3c and Supplementary Figure S7b). Thus, the YugJ structures in complex with NADP form more optimized interactions with NADP and Ni^2+^ in a narrower interdomain cleft than the NADP-free structures. Based on these findings, we propose that YugJ shifts its dynamic equilibrium toward a closed conformation upon NADPH binding.

### 2.4. Putative Substrate-Binding Site of YugJ

**Figure 4:**
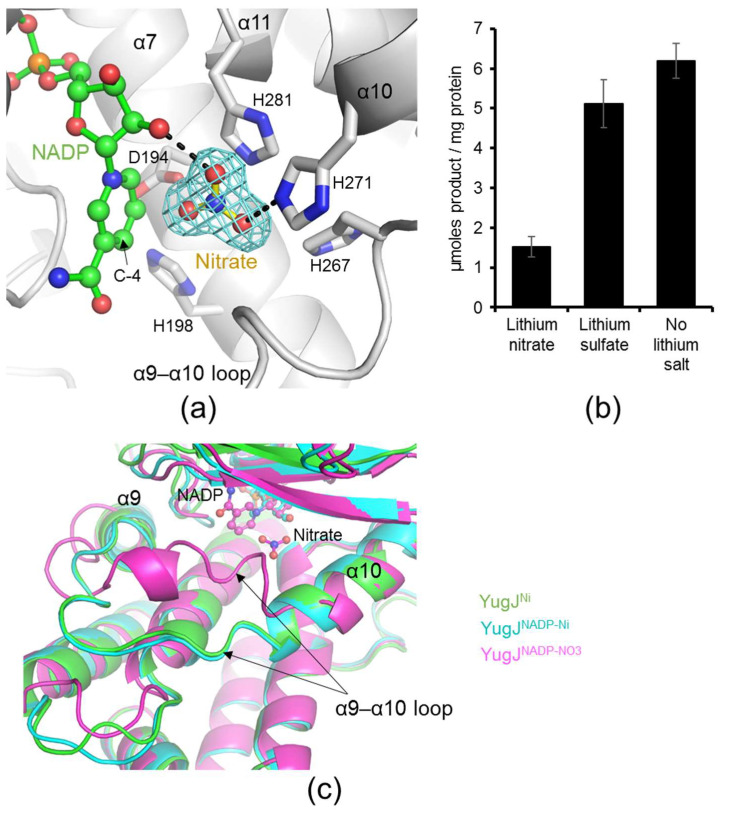
Nitrate binding of YugJ and inhibitory activity of nitrate on YugJ catalysis. (**a**) Putative substrate-binding site of YugJ at or near the nitrate ion in the YugJ^NADP-NO3^ structure. YugJ is depicted as gray ribbons. The NADP cofactor and nitrate ion are shown as ball-and-stick models, as shown in Figure 3b. The electron density of the nitrate ion (3σ in the Fo−Fc omit map) is represented by cyan meshes. The nitrate-binding and metal-coordinating residues of YugJ are shown as gray sticks on the YugJ structure. The hydrogen bonds of the nitrate ion with YugJ residues and NADP are represented by black dotted lines. (**b**) Nitrate-specific inhibition of YugJ activity. The HMF reduction activity of YugJ was determined in the presence of lithium nitrate or lithium sulfate. (**c**) Closed state of the α9–α10 loop lid in the YugJ^NADP-NO3^ structure (magenta ribbons) in contrast to an open state in the YugJ^NADP-Ni^ (cyan ribbons) and YugJ^Ni^ (green ribbons) structures.

Because YugJ accommodates NADP and metal cofactors in the interdomain cleft, the cleft highly likely functions as an active site that binds and transforms the substrate. To provide insights into the substrate-binding site of YugJ, we carefully analyzed the electron density of the YugJ structures in the interdomain cleft. Notably, in the YugJ^NADP-NO3^ crystal structure, a three-pointed star-like electron density was observed near the nicotinamide ring of NADP in the interdomain cleft (Figure 4a). The extra density is most likely derived from a nitrate ion, given that nitrate ions were used for YugJ^NADP-NO3^ crystallization and resemble a three-pointed star. Accordingly, an additional three-pointed density was not observed in the YugJ^Ni^ or YugJ^NADP-Ni^ structure obtained in the absence of nitrate. Therefore, the three-pointed star-like electron density was modeled as a nitrate ion in the YugJ^NADP-NO3^ structure.

In the YugJ^NADP-NO3^ structure, the nitrate ion is enclosed by NADP and YugJ residues from the α9–α10 loop and α10 and α11 helices. The nitrate ion forms hydrogen bonds with the nicotinamide-linked ribose moiety of NADP and the imidazole ring of the YugJ His271 residue. Noticeably, the oxygen atom of the nitrate ion is located in close proximity to the C-4 atom of the nicotinamide moiety that undergoes oxidation upon substrate reduction, suggesting that the oxygen atom of the nitrate ion positionally mimics that of the aldehyde substrate. Interestingly, other aldehyde reductase structures have been reported to accommodate various chemicals at or near the nitrate-binding site of the YugJ^NADP-NO3^ structure [12,14,45]. Based on these observations, we propose that the nitrate-binding site is used by YugJ to recognize the aldehyde substrate. To confirm this proposal, a competitive inhibition assay was performed using nitrate (Figure 4b). The HMF-reducing activity of YugJ decreased by ~75% in the presence of lithium nitrate, suggesting that nitrate ions compete with HMF to bind the active site of YugJ. The inhibitory effect seems to be specific to nitrate, given that lithium sulfate addition did not significantly reduce YugJ activity.

Noticeably, YugJ exhibits substantial structural differences at the α9–α10 loop, which neighbors the nitrate ion in the YugJ^NADP-NO3^ structure (Figure 4c). YugJ^NADP-NO3^ locates the α9–α10 loop closer to the active site than the YugJ^Ni^ and YugJ^NADP-Ni^ structures and forms an additional α-helix in the middle of the α9–α10 loop. These structural changes seem to be induced by the YugJ^NADP-NO3^-specific interactions of the α9–α10 loop residues, Trp264 and Arg261, with the nitrate ion and β5–β6 loop, respectively. As a result of the structural rearrangement, the α9–α10 loop completely covers the nitrate ion in the YugJ^NADP-NO3^ structure and induces closure of the active site. In contrast, the α9–α10 loop of the YugJ^Ni^ and YugJ^NADP-Ni^ structures adopts an open conformation that increases the accessibly of the active site. Given that nitrate inhibits the HMF-reducing activity of YugJ presumably via a competitive inhibition mechanism, we propose that the α9–α10 loop functions as a lid that closes the active site upon substrate binding for substrate stabilization.

Nitrate binding appears to induce structural changes in NADP(H) in addition to the YugJ α9–α10 loop. In the YugJ^NADP-NO3^ structure, the nicotinamide ring of NADP adopts a rigid structure, interacting with the nitrate ion (Figure 4a and Supplementary Figure S6a). On the other hand, in the nitrate-less YugJ^NADP-Ni^ structure, the nicotinamide ring of NADP is disordered, although similar conformations are observed for the YugJ Asp102, Asn147, and Gly149 residues that interact with nicotinamide in the YugJ^NADP-NO3^ structure (Figure 3b and Supplementary Figure S6b). Thus, we propose that NADP(H) nicotinamide in YugJ prefers high structural dynamics for substrate recognition and becomes rigid upon substrate binding.

**Figure 5:**
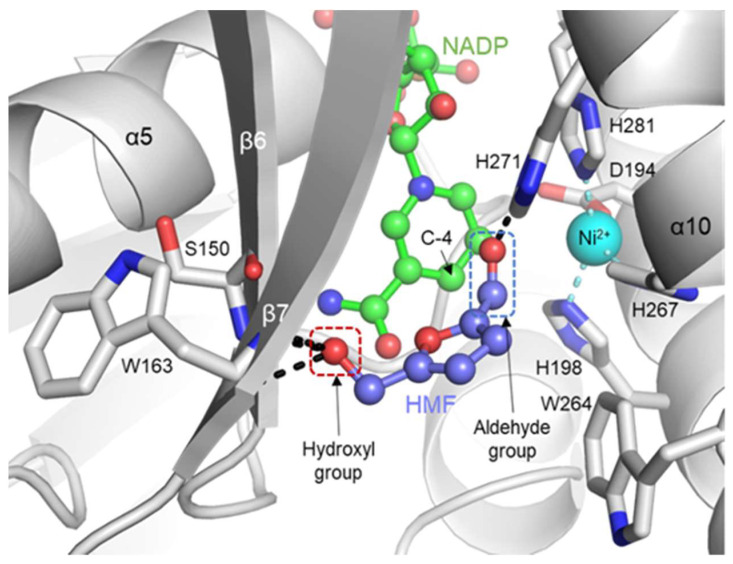
YugJ-HMF complex model based on molecular docking. HMF was docked on the YugJ-NADP-Ni^2+^ model. HMF and NADP are represented by light blue and green ball-and-stick models, respectively. The Ni^2+^ ion is represented by a cyan sphere. The HMF-binding residues and Ni^2+^-coordinating residues of YugJ are shown as gray sticks on the YugJ structure (gray ribbons). The hydrogen bonds of HMF with YugJ residues are represented by black dotted lines. The aldehyde and hydroxyl groups of HMF are indicated by blue and red dotted boxes, respectively.

To further investigate the substrate-binding mechanism of YugJ, we modeled the HMF-bound YugJ structure through in silico molecular docking [46]. In the complex model, HMF is found near the nicotinamide moiety of NADP in the interdomain cleft, and the aldehyde group of HMF is oriented toward the C-4 atom of the nicotinamide ring and the Ni^2+^ ion (Figure 5). Notably, the aldehyde group of HMF sterically clashes with the nitrate ion from the overlaid YugJ^NADP-NO3^ structure (Supplementary Figure S8). In the YugJ-HMF model, YugJ residues clasp and stabilize the termini of HMF (aldehyde and hydroxyl groups), properly orienting HMF for reduction. The His271 residue from the CTD α10 helix forms a hydrogen bond with the aldehyde group of HMF. The contribution of His271 to substrate binding has been reported in previous mutation studies on an *E. coli* homolog of YugJ [9,47]. The Ser150 and Trp163 residues from the NTD β6 and β7 strands, respectively, directly interact with the hydroxyl group of HMF, explaining why furfural, which lacks the hydroxyl group, is less efficiently reduced by YugJ than HMF.

**Figure 6:**
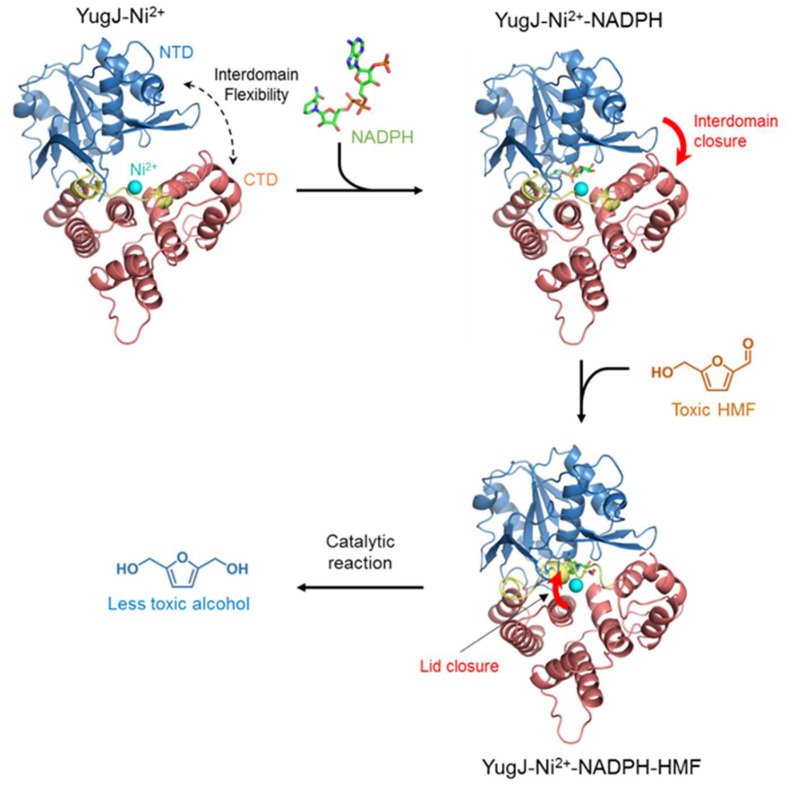
A model for YugJ binding to NADPH and HMF. The YugJ NTD and CTD are shown as blue and red ribbons, respectively, and the α9–α10 loop lid in the CTD is highlighted in yellow. The NADPH and Ni^2+^ cofactors are depicted as green sticks and a cyan sphere, respectively. NADPH and HMF binding-induced structural changes in YugJ are represented by thick red arrows.

In conclusion, we identified *B. subtilis* YugJ as an NADPH-dependent furan aldehyde reductase based on structural and biochemical studies. Our comparative analysis of the YugJ structures allows us to propose initial catalytic events that occur during YugJ-mediated HMF reduction (Figure 6). First, NADPH is inserted into the interdomain cleft of YugJ, limiting the interdomain dynamics of YugJ into a closed conformation. Next, the HMF substrate binds the active site near the NADPH and metal cofactors and is stabilized via closure of the α9–α10 loop lid for subsequent product formation. Our findings and proposal will provide a structural basis for understanding the aldehyde reduction mechanism of YugJ and designing enzymes with improved furan aldehyde reduction activity.

## 3. Materials and Methods

### 3.1. Construction of a YugJ Expression Plasmid

The open reading frame that encodes the full-length YugJ protein (GenBank: EFG92987.1; residues 1–387) was amplified by PCR from the genomic DNA of *B. subtilis* subsp. *spizizenii* using primers containing the *Bam*HI or *Sal*I recognition sequence. The PCR product was digested using the *Bam*HI and *Sal*I restriction enzymes and ligated into a pET49b plasmid that had been modified to express the recombinant protein in fusion with a His_6_ tag and a thrombin cleavage site at the N-terminus. The ligation product was transformed into *E. coli* DH5α cells. The nucleotide sequence of the *YugJ* gene in the pET49b plasmid was verified by DNA sequencing.

### 3.2. Expression and Purification of the YugJ Protein

The YugJ expression plasmid was transformed into *E. coli* BL21 (DE3) cells for YugJ overexpression. Transformant cells were grown at 37 °C in LB medium until the optical density at 600 nm reached 0.6–0.8. The YugJ protein was overexpressed in the presence of 1 mM isopropyl-β-D-1-thiogalactopyranoside at 18 °C overnight. The *E. coli* cells were harvested by centrifugation and lysed by sonication in a solution containing 50 mM Tris, pH 8.0, 200 mM NaCl, 5 mM β-mercaptoethanol, and 1 mM phenylmethanesulfonyl fluoride. The cell lysate was cleared by centrifugation, and the resultant supernatant was incubated with Ni-NTA resin (Qiagen, Venlo, Netherlands) to purify the His_6_-tagged YugJ protein via affinity chromatography. The YugJ protein was eluted from Ni-NTA resin using 250 mM imidazole and dialyzed against 20 mM Tris, pH 8.0, and 5 mM β-mercaptoethanol. The resulting protein was incubated with thrombin at 18 °C for 24 h to remove the N-terminal His_6_ tag. The tag-free YugJ protein was loaded onto a Mono Q 10/100 GL column (GE Healthcare, Chicago, IL, USA) for further purification by anion-exchange chromatography and eluted from the Mono Q resin using a 0–500 mM NaCl gradient. The oligomeric state of the purified YugJ protein was analyzed by gel-filtration chromatography using a Superdex 200 10/300 column (GE Healthcare, Chicago, IL, USA) in 20 mM Tris, pH 8.0, 150 mM NaCl, and 5 mM β-mercaptoethanol.

### 3.3. Crystallization and X-ray Diffraction

YugJ crystallization conditions were screened with JCSG Core Suites (Qiagen, Venlo, Netherlands) and optimized by the sitting-drop vapor-diffusion method at 18 °C using YugJ protein in 20 mM Tris, pH 8.0, 200 mM NaCl, and 5 mM β-mercaptoethanol. YugJ^Ni^ crystals were generated by mixing 0.5 μL of YugJ protein (12.7 mg/mL) with 0.5 μL of a well solution containing 20% PEG 6000 and 0.1 M Tris, pH 8.0, and then equilibrating the resultant 1-μL drop against 500 μL of the well solution. To obtain YugJ^NADP-NO3^ crystals, YugJ protein (18.3 mg/mL) was mixed with NADP at a molar ratio of 1:3, and the resulting mixture was equilibrated to a well solution containing 18% PEG 3350, 0.1 M MES, pH 6.5, and 0.3 M ammonium nitrate. To generate YugJ^NADP-Ni^ crystals, YugJ crystals were first obtained in a drop containing 0.5 μL of YugJ protein (13.6 mg/mL) and 0.5 μL of a well solution (14% PEG 8000, 0.2 M calcium acetate, and 0.1 M MES, pH 6.5) and were then soaked in a solution containing 3 mM NADP and 3 mM HMF.

A YugJ crystal was briefly soaked in a cryoprotectant solution containing 25% glycerol or 25% ethylene glycol and flash-cooled under a nitrogen cryostream. X-ray diffraction was performed at the Pohang Accelerator Laboratory (Pohang, Korea), and diffraction data were reduced and scaled using the HKL2000 package [48]. To verify the presence of Ni^2+^ ions in the YugJ crystal, the X-ray fluorescence emission spectrum was obtained from the YugJ crystal. Data collection statistics are summarized in Table 1.

### 3.4. Structure Determination

The YugJ^Ni^ structure was determined by molecular replacement with the Phaser program using the structure of *T. maritima* butanol dehydrogenase A (PDB ID 1VLJ) as a search model [49]. Model building and refinement were performed using the Coot and Phenix programs, respectively, to yield the final structure of YugJ^Ni^ [50,51]. The YugJ^NADP-NO3^ and YugJ^NADP-Ni^ structures were solved by molecular replacement using the YugJ^Ni^ structure as a search model. The final YugJ^NADP-Ni^ and YugJ^NADP-NO3^ structures were obtained via iterative cycles of model building and refinement [50,51]. The atomic coordinates and the structure factors for the YugJ^Ni^, YugJ^NADP-Ni^, and YugJ^NADP-NO3^ structures (PDB ID 7W9X, 7W9Y, and 7W9Z, respectively) have been deposited in the Protein Data Bank.

Structure refinement statistics indicate that the three structures are overall of high quality (Table 1). However, the YugJ^NADP-Ni^ structure has two Ramachandran outliers at the Ala141 residues from chains B and D. The two YugJ^NADP-Ni^ residues are located at the boundary between the favored region and outlier region of the Ramachandran plot, as observed for the Ala141 residues from chains A and C. Moreover, these four residues exhibit similar conformations. Notably, the YugJ^Ni^ structure displays a higher average B-factor (48.7 Å^2^) than the YugJ^NADP-Ni^ (29.4 Å^2^) and YugJ^NADP-NO3^ (22.2 Å^2^) structures, presumably due to lower resolution and a more disordered crystal lattice. However, the quality of the data collected for YugJ^Ni^ is similar to those of YugJ^NADP-Ni^ and YugJ^NADP-NO3^.

### 3.5. Measurement of the Catalytic Activity of YugJ

The enzymatic activity of YugJ was evaluated using demetallized YugJ protein. To obtain metal-free YugJ protein, the purified recombinant YugJ protein was treated with a solution containing 1 mM EDTA and 100 mM sodium acetate, pH 5.0, at 4 °C for 2 h and subsequently diluted ~90-fold using a solution containing 20 mM Tris, pH 8.0, and 150 mM NaCl [52]. The metal-free YugJ protein (12 μM) was preincubated with divalent metal ions (120 μM) prior to the measurement of catalytic activity.

To investigate the substrate specificity of YugJ, 1 μg of the Ni^2+^-bound YugJ protein was reacted with 3 mM aldehyde substrate candidate (HMF, furfural, butyraldehyde, propionaldehyde, acetaldehyde, isobutyraldehyde, glyoxal, or methylglyoxal) in the presence of 0.2 mM NADPH in 200 μL of a reaction solution, containing 50 mM HEPES, pH 7.4, and 150 mM NaCl, at 23 °C. The catalytic activity of YugJ was also assessed for 2,5-bis(hydroxymethyl)furan alcohol in the presence of 0.2 mM NADP in the reaction solution (pH 7.4) at 23 °C. The pH dependence of YugJ-mediated HMF reduction was determined by incubating 1 μg of Ni^2+^-bound YugJ with 3 mM HMF and 0.2 mM NADPH in a 200-μL solution, containing 150 mM NaCl and 50 mM buffer at different pH values (pH 6.0, 6.5, 7.0, 7.5, 8.0, 8.5, 9.0, and 9.5) at 23 °C. To evaluate the metal specificity of YugJ, 1 μg of the YugJ protein preincubated with different metal ions was reacted with 3 mM HMF and 0.2 mM NADPH in 200 μL of the reaction solution (pH 7.4) at 23 °C. To determine the temperature dependence of YugJ-mediated HMF reduction, 1 μg of the Ni^2+^-bound YugJ protein was reacted with 3 mM HMF and 0.2 mM NADPH at temperatures between 16 and 58 °C in 200 μL of the reaction solution (pH 7.4). The reducing cofactor dependence of YugJ-mediated HMF reduction was determined by mixing 1 μg of the Ni^2+^-bound YugJ protein with 0.2 mM NADPH or NADH in the presence of 3 mM HMF in the reaction solution (pH 7.4) at 23 °C. The inhibition of YugJ-mediated HMF reduction by nitrate was addressed by incubating 360 mM lithium nitrate or lithium sulfate with 1 μg of Ni^2+^-bound YugJ, 3 mM HMF, and 0.2 mM NADPH in 200 μL of the reaction solution (pH 7.4) at 23 °C. The catalytic activity of YugJ was determined by measuring the absorbance at 340 nm using an EPOCH spectrophotometer (BioTek, Winooski, VT, USA).

To derive the kinetic parameters of YugJ, 1 μg of the Ni^2+^-bound YugJ protein was reacted with serially diluted HMF (0.3–40 mM) in the presence of 0.2 mM NADPH in 200 μL of the reaction solution (pH 7.4) at 23 °C. The absorbance at 340 nm was measured in a continuous manner. The enzymatic data were fitted to the Michaelis–Menten equation using the Prism 5 program (GraphPad, CA, USA).

### 3.6. In Silico Molecular Docking Analysis

The structure of YugJ in complex with HMF was modeled via in silico molecular docking. The coordinates of HMF were obtained in the SDF format from the PubChem database [53]. The coordinate files of the HMF ligand and YugJ protein were converted to the PDBQT format using the AutoDock Tools package. Next, the HMF ligand was docked on the YugJ protein using the AutoDock Vina program [46]. The docked ligands were analyzed based on binding energy. The YugJ-HMF complex model was graphically represented using the PyMOL program.

## Figures and Tables

**Table 1:** Crystallographic statistics of the YugJ structures.

	YugJ^Ni^	YugJ^NADP-Ni^	YugJ^NADP-NO3^
**Data collection**			
Space group	P2_1_2_1_2_1_	P1	P2_1_
Cell parameters			
a (Å)	62.66	66.04	56.51
b (Å)	114.52	69.64	67.69
c (Å)	118.74	87.61	102.37
α (°)	90	92.29	90
β (°)	90	91.03	100.15
γ (°)	90	90.37	90
Wavelength (Å)	1.0000	0.9793	1.0000
Resolution (Å)	30.00–2.15	30.00–1.93	30.00–1.65
Highest resolution (Å)	2.19–2.15	1.96–1.93	1.68–1.65
No. uniquere flections	47,100 (2297) ^a^	112,718 (5537) ^a^	91,189 (4474) ^a^
R_merge_ (%) ^b^	6.8 (50.0) ^a^	5.5 (35.1) ^a^	7.7 (44.1) ^a^
R_meas_ (%) ^c^	7.6 (55.8) ^a^	7.8 (49.7) ^a^	9.0 (51.7) ^a^
CC_1/2_^d^	0.997 (0.860) ^a^	0.997 (0.740) ^a^	0.994 (0.879) ^a^
I/sigma(I)	32.6 (5.0) ^a^	19.1 (2.8) ^a^	27.4 (4.2) ^a^
Completeness (%)	99.9 (100.0) ^a^	96.7 (95.3) ^a^	99.6 (99.0) ^a^
Redundancy	4.9 (5.0) ^a^	2.0 (1.9) ^a^	3.7 (3.7) ^a^
Wilson B-factor (Å^2^)	33.4	22.1	16.4
**Refinement**			
Resolution (Å)	30.00–2.15	30.00–1.93	30.00–1.65
No. of reflections (work)	44,658	106,897	86,549
No. of reflections (test)	2376	5775	4572
R_work_ (%) ^e^	20.3	17.0	17.4
R_free_ (%) ^f^	24.1	20.8	19.7
Estimated coordinate error (Å)	0.25	0.19	0.16
No. atoms			
Protein	5717	11,878	5897
Metals	2	4	-
NADP	-	160	96
Nitrate	-	-	16
Water	116	792	605
Average B-value (Å^2^)			
Protein	49.0	29.2	21.6
Metals	51.1	26.8	-
NADP	-	22.8	13.8
Nitrate	-	-	23.3
Water	37.0	32.5	29.7
RMSD bonds (Å)	0.008	0.007	0.006
RMSD angles (°)	0.864	0.851	0.899
Ramachandran ^g^ (favored)	97.4%	97.5%	96.8%
Ramachandran ^g^ (outliers)	0.0%	0.1%	0.0%
PDB ID	7W9X	7W9Y	7W9Z

^a^ Numbers in parentheses were calculated from data for the highest resolution shell. ^b^ R_merge_ = Σ_hkl_Σ_i_|I_i_(hkl)—<I(hkl)> |/Σ_hkl_Σ_i_ I_i_(hkl); ^c^ R_meas_ = Σ_hkl_{N(hkl)/[N(hkl)—1]}^1/2^ Σ_i_ | I_i_(hkl)—<I(hkl)>|/Σ_hkl_Σ_i_ I_i_(hkl); ^d^ Correlation coefficient between intensities from random half-data sets. ^e^ R_work_ = Σ||F_obs_|-|F_calc_||/Σ|F_obs_| where F_calc_ and F_obs_ are the calculated and observed structure factor amplitudes, respectively. ^f^ R_free_ = as described for R_work_, except that 5% of the total reflections were selected at random and omitted from refinement. ^g^ Calculated using MolProbity (http://molprobity.biochem.duke.edu, accessed on 11 December 2021).

## Data Availability

The atomic coordinates and the structure factors for YugJ (PDB ID 7W9X, 7W9Y, and 7W9Z) have been deposited in the Protein Data Bank (www.pdb.org, deposited on 11 December 2021).

## Supplementary Materials

The following supporting information can be downloaded at: https://www.mdpi.com/article/10.3390/ijms23031882/s1. Reference [54] is cited in the supplementary materials.

## Abbreviations

AAOR, NAD(H)/NADP(H)-dependent aldehyde/alcohol oxidoreductase; CTD, C-terminal domain; HMF, 5-hydroxymethylfurfural; NTD, N-terminal domain; YugJ^NADP-Ni^, YugJ in complex with NADP and Ni^2+^; YugJ^NADP-NO3^, YugJ in complex with NADP and nitrate ions; YugJ^Ni^, YugJ in complex with Ni^2+^.
