# Supplementary material for: Structural and Biochemical Analysis of the Furan Aldehyde Reductase YugJ from Bacillus subtilis
Source: Int J Mol Sci. 2022 Feb 8;23(3):1882. doi: 10.3390/ijms23031882 (PMC8836905; doi:10.3390/ijms23031882)
Supplement: Supplementary file 1 [file ijms-23-01882-s001.zip › ijms-1558692-supplementary.pdf]

Supplementary Figure S1

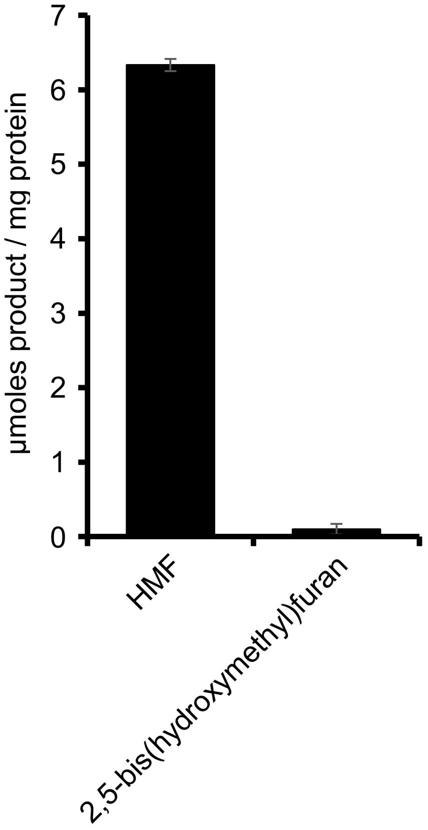

Supplementary Figure S1. Enzymatic function of YugJ as an aldehyde reductase but not as an alcohol dehydrogenase. The catalytic activities of recombinant YugJ protein toward HMF and 2,5-bis(hydroxymethyl)furan were determined in the presence of NADPH and NADP, respectively, at pH 7.4 with Ni^{2+} ions.

Supplementary Figure S2

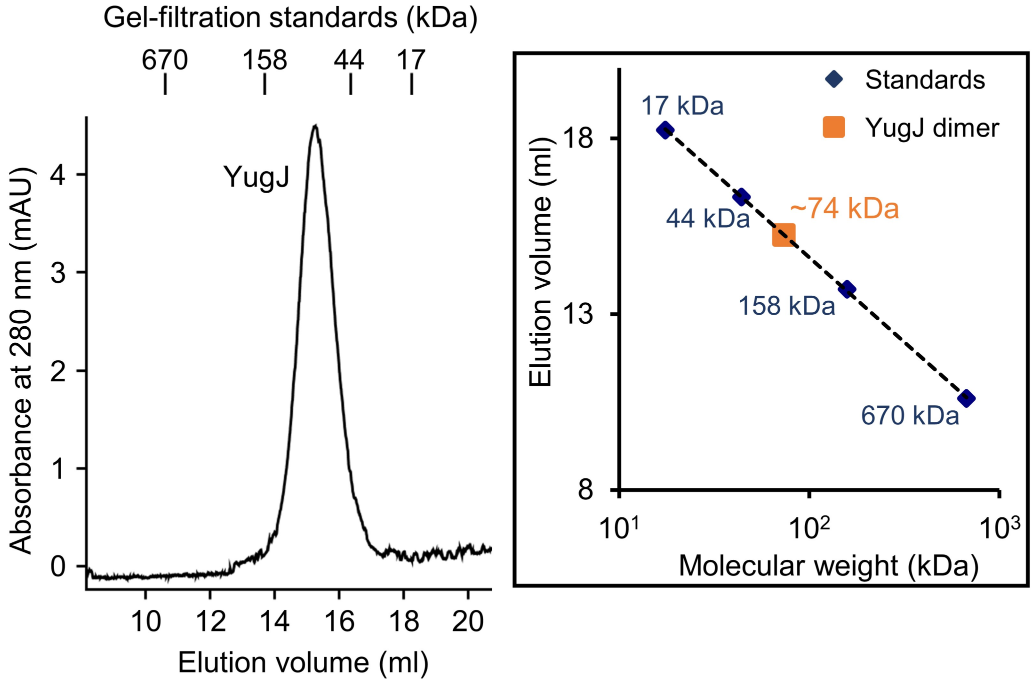

Supplementary Figure S2. YugJ dimer in solution. In gel-filtration chromatography, the YugJ protein was eluted as a dimer with a single peak. The apparent molecular weight of YugJ (~74 kDa) was estimated based on the elution volumes of gel-filtration standards.

Supplementary Figure S3

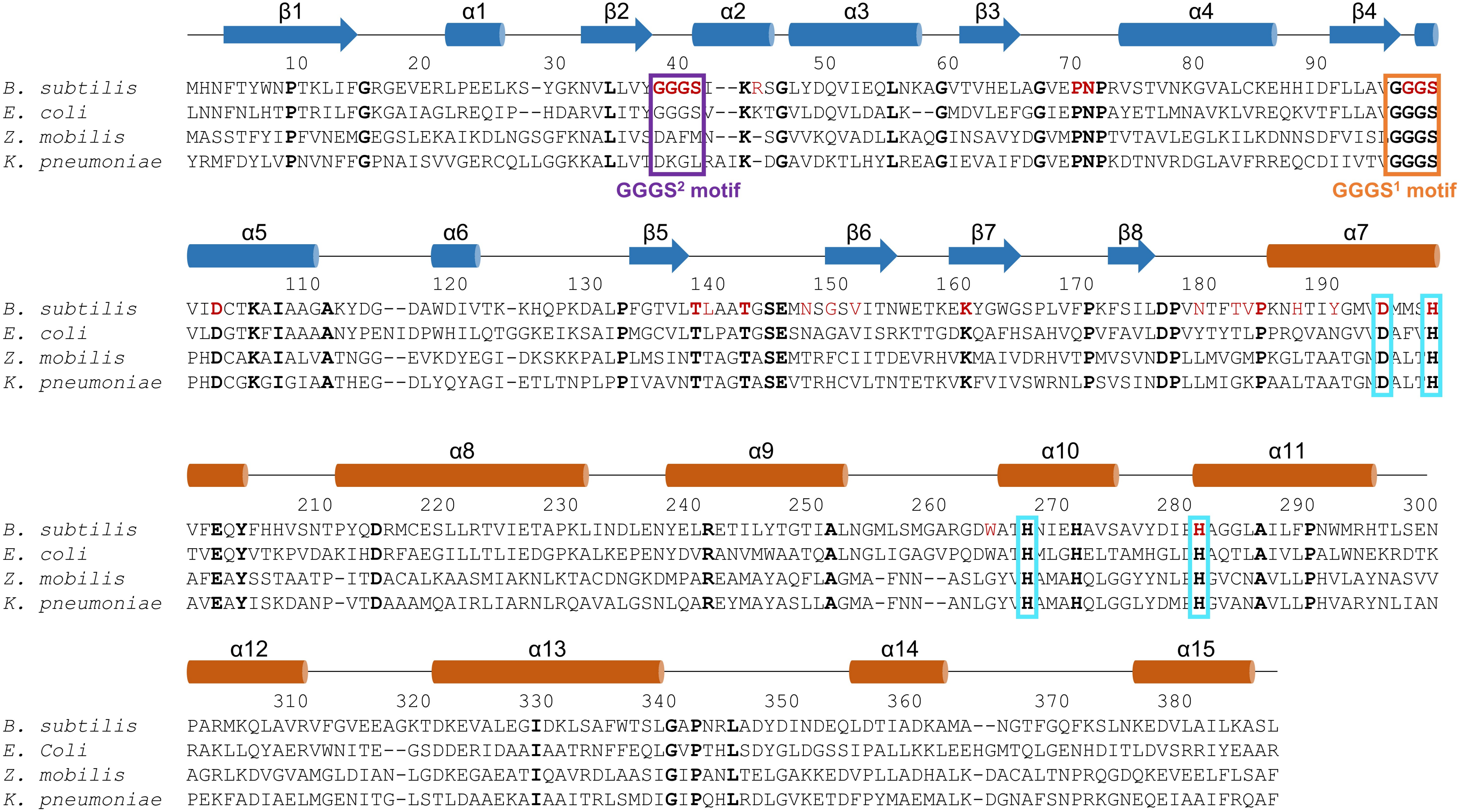

Supplementary Figure S3. Sequence alignment of YugJ and its structural homologs [aldehyde reductase YqhD from *E. coli* K-12 (37% sequence identity), iron-dependent alcohol dehydrogenase 2 from *Z. mobilis* ZM4 (27% sequence identity), and 1,3-propanediol dehydrogenase from *K. pneumoniae* (27% sequence identity)] using the PROMALS3D server [54]. The secondary structures of YugJ are shown as cylinders (α-helices) and arrows (β-strands) in domain-specific colors (NTD, blue; CTD, red) above the amino acid sequence of YugJ. Conserved amino acids are shown in bold font, and the NADP-binding residues of YugJ are colored red. Metal ion-coordinating residues are indicated by cyan boxes. The GGGS[1] and GGGS[2] motifs that are involved in NADP cofactor binding are indicated by orange and purple boxes, respectively.

Supplementary Figure S4

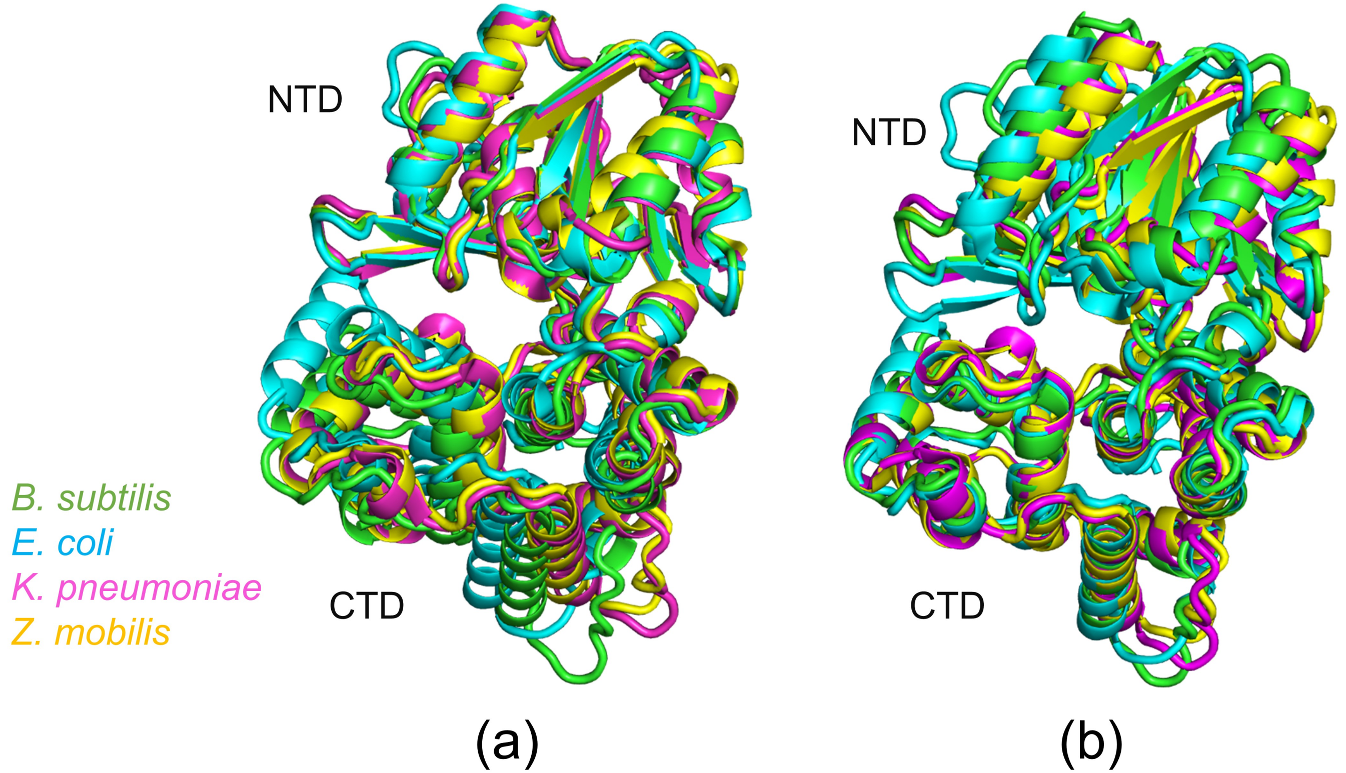

Supplementary Figure S4. Structural similarity of YugJ with other group III AAORs. The structures of *E. coli* YqhD (PDB ID 1OJ7; cyan ribbons), *K. pneumoniae* 1,3-propanediol dehydrogenase (PDB ID 3BFJ; magenta ribbons), and *Z. mobilis* alcohol dehydrogenase 2 (PDB ID 3OWO; yellow ribbons) are overlaid on the *B. subtilis* $YugJ^{Ni}$ structure (green ribbons) using the NTDs (a) or CTDs (b). The overall domain structures are similar. However, NTD-CTD orientations differ due to interdomain flexibility.

Supplementary Figure S5

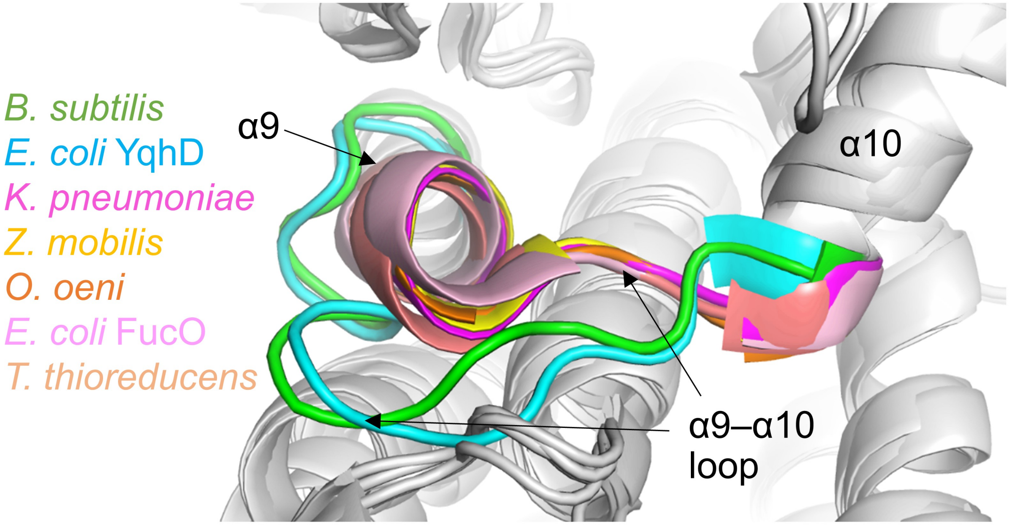


```
                    252          265
                     ▼            ▼
B. subtilis         LNGMLSMGARGDWA
E. coli YqhD        LNGLIGAGVPQDWA
K. pneumoniae       GMA-FNN--ASLGY
Z. mobilis          GMA-FNN--ANLGY
O. oeni             GMA-FNN--ANLGY
E. coli FucO        GMG-FSN--VGLGL
T. thioreducens     GIA-FLN--ARLGL
```

Supplementary Figure S5. Structural comparison of the α9–α10 loops from *B. subtilis* YugJ and other group III AAORs. The α9–α10 loops from the structures of *B. subtilis* YugJ^{Ni}, *E. coli* YqhD (PDB ID 1OJ7), *K. pneumoniae* 1,3-propanediol dehydrogenase (PDB ID 3BFJ), *Z. mobilis* alcohol dehydrogenase 2 (PDB ID 3OWO), *Oenococcus oeni* alcohol dehydrogenase (PDB ID 4FR2), *E. coli* lactaldehyde reductase FucO PDB ID 5BR4), and *Thermococcus thioreducens* alcohol dehydrogenase (PDB ID 6C75) are colored green, cyan, magenta, yellow, orange, pink, and salmon, respectively (left panel). The amino acid sequences of the colored regions in the left panel are aligned (right panel).

Supplementary Figure S6

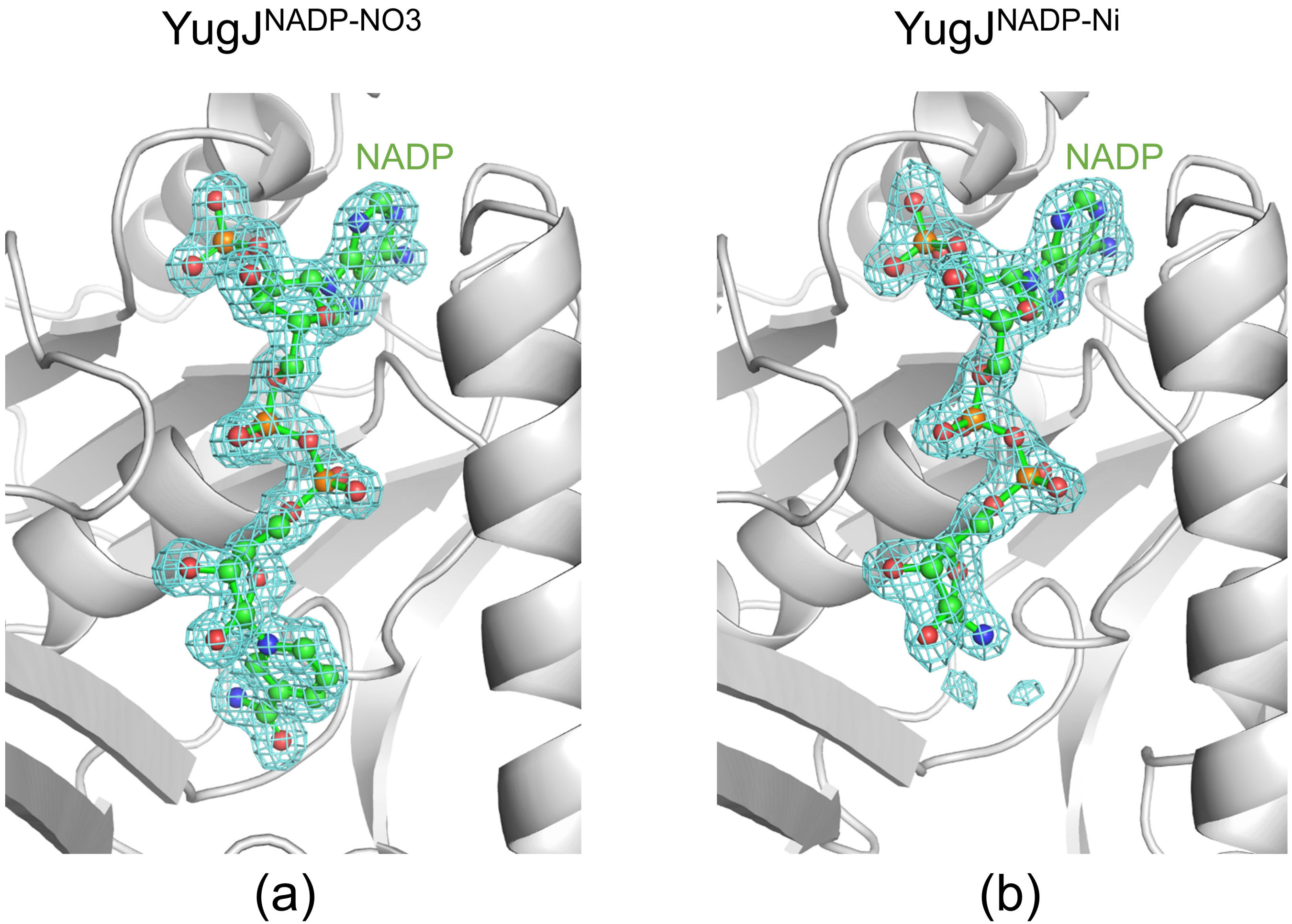

Supplementary Figure S6. Electron density of NADP in the YugJ$^{NADP\text{-}NO3}$ (a) and YugJ$^{NADP\text{-}Ni}$ (b) structures. YugJ and NADP are depicted as gray ribbons and a ball-and-stick model, respectively. The electron density of NADP (3σ in the Fo−Fc omit map) is represented by cyan meshes.

Supplementary Figure S7

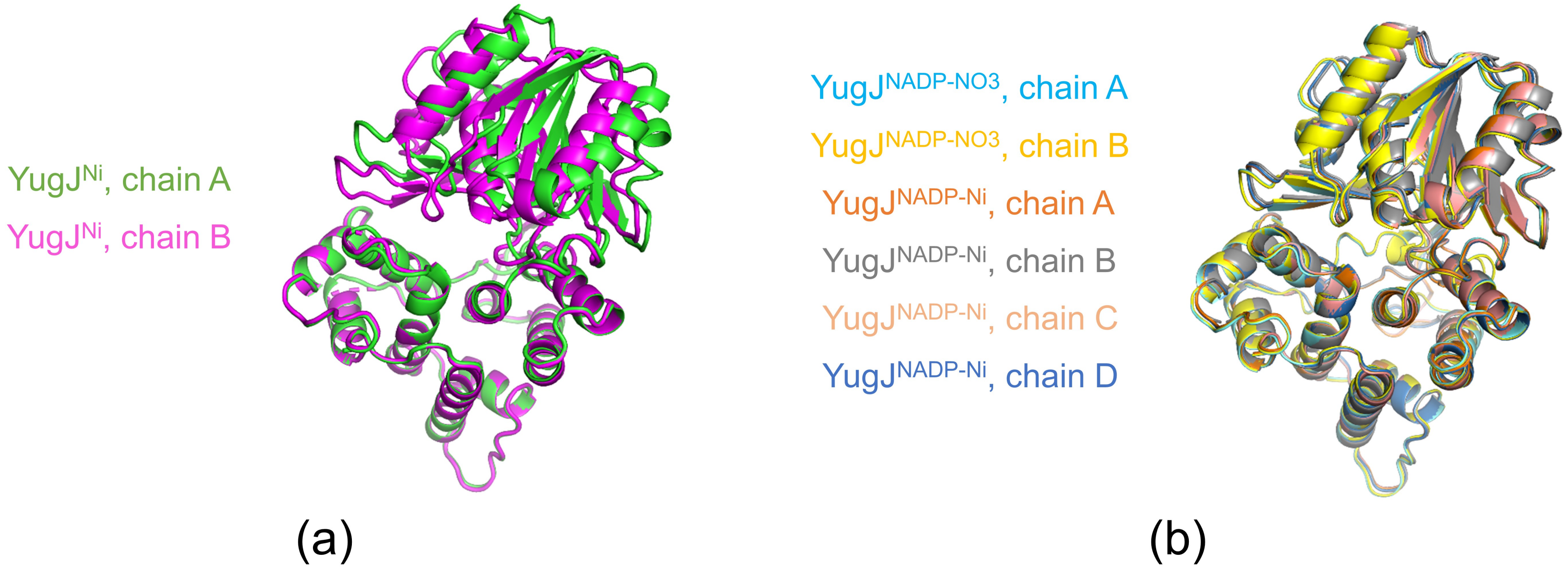

Supplementary Figure S7. NADP binding-dependent change in the interdomain flexibility of YugJ. The YugJ structures are superimposed using the CTDs as a reference point. (a) Interdomain flexibility of the NADP-free YugJ protein. YugJ^{Ni} chains A (green ribbons) and B (magenta ribbons) adopt open and closed conformations, respectively. (b) Similar interdomain organization of the NADP-bound YugJ structures. Two YugJ$^{NADP-NO3}$ chains (chain A, cyan; chain B, yellow) and four YugJ$^{NADP-Ni}$ chains (chain A, orange; chain B, gray; chain C, salmon; chain D, blue) adopt closed conformations.

Supplementary Figure S8

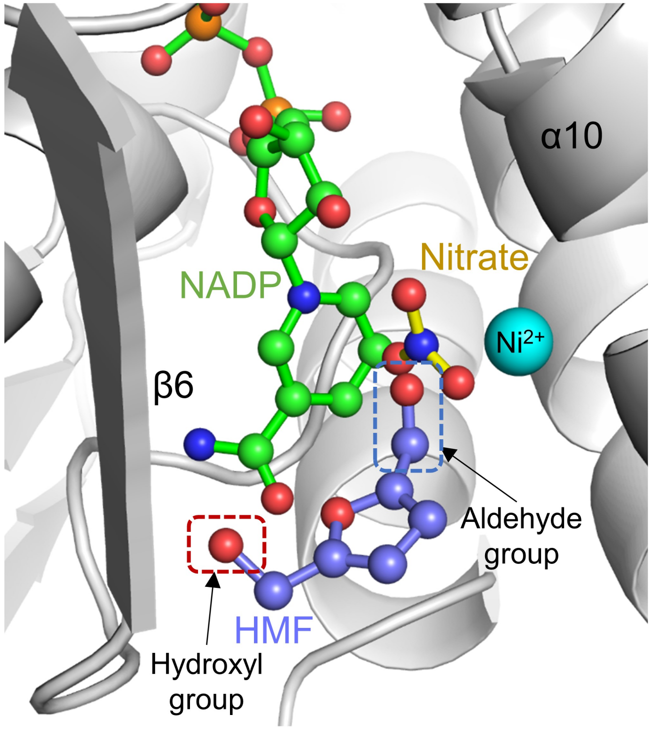

Supplementary Figure S8. HMF docked in the active site of YugJ at or near the nitrate-binding region. In the quaternary YugJ-HMF-NADP-Ni^{2+} model, the HMF substrate (light blue ball-and-stick model) is located near the nicotinamide ring of NADP (green ball-and-stick model) and the Ni^{2+} ion (cyan sphere) and positionally overlaps with the nitrate ion (yellow ball-and-stick model) from the $YugJ^{NADP\text{-}NO3}$ structure.
